# Gomme du visage revelant une syphilis congenitale tardive

**DOI:** 10.11604/pamj.2013.16.29.3360

**Published:** 2013-09-29

**Authors:** Amal Taghy, Badreddine Hassam

**Affiliations:** 1 Service de Dermatologie-Vénérologie, CHU Ibn Sina, Maroc / Faculté de Médecine et de Pharmacie, Med V Souissi, Rabat, Maroc

**Keywords:** syphilis congénitale, gomme syphilitique, congenital syphilis, gumma

## Image en medicine

La syphilis congénitale continue de poser un problème de santé dans les pays en voie de développement. Devenue rare dans les pays industrialisés dans les années 1990 grâce à l'avènement de la pénicillothérapie, le dépistage systématique et la prévention chez la femme enceinte, sa résurgence depuis l'année 2000 est confirmée notamment chez les homosexuels et les sujets infectés par le VIH. La transmission mère-enfant est d'autant plus importante que la syphilis maternelle soit récente et que l'on approche de l'accouchement. À l'opposé de la syphilis congénitale néonatale, la syphilis congénitale tardive qui s'exprime entre l'âge de 20 à 30 ans en l'absence de traitement adéquat, est exceptionnelle et considérée comme l'équivalent congénital de la syphilis tertiaire. Elle est asymptomatique dans 40 % des cas, mais peut se compliquer d'atteintes oculaires, neurologiques, cochléaires, ostéoarticulaires, rarement de gommes cutanéo-muqueuses ainsi que d'autres atteintes viscérales. Les tests sérologiques (TPHA, VDRL et FTA-abs) demeurent les examens de choix pour le dépistage de la syphilis congénitale. Bien qu'il n'y a pas de consensus thérapeutique qui varie selon le tableau clinique, Le traitement repose sur la pénicilline G en intramusculaire à doses progressives afin d'éviter la réaction d'Herxheimer. La réponse thérapeutique est appréciée cliniquement et sérologiquement. Les lésions de la syphilis tardive régressent plus lentement et incomplètement et les manifestations cliniques sont en rapport avec les réactions tissulaires induites par le tréponème plutôt qu'avec sa prolifération. Il s'agit de la gomme syphilitique (nécrose acellulaire). Les tests tréponémiques (TPHA et FTA) semblent rester aussi indéfiniment positifs. La prévention repose sur le dépistage systématique et le traitement précoce des mères atteintes. Nous rapportons un cas d'une jeune fille de 18 ans, admise pour tuméfaction sous-cutanée de la joue droite, ovalaire, évoluant depuis 4 mois, mesurant cinq centimètres de grand axe, rouge-cuivrée, indolore, non prurigineuse, initialement de consistance ferme puis devenant fluctuante à la palpation. Le reste de l'examen cutanéo-muqueux et systémique était normal. Une sérologie syphilitique avait objectivé un taux sanguin de TPHA à 1/320 et VDRL à 1/4. Ces taux étaient négatifs dans le liquide céphalorachidien avec une protéinorrachie à 0,14 g/L et moins de 2 éléments blancs par mm3. Le diagnostic de syphilis congénitale tardive était retenu devant l'absence de facteurs de risque chez la patiente et les antécédents de syphilis chez la mère et une biopsie cutanée avait confirmé le diagnostic. La patiente était traitée par benzathine benzylpénicilline; 2,4 millions par semaine pendant 6 semaines avec évolution clinique lentement favorable et persistance de la sérologie syphilitique positive avec toutefois, une baisse du VDRL 4 fois moindre que le taux initial.

**Figure 1:**
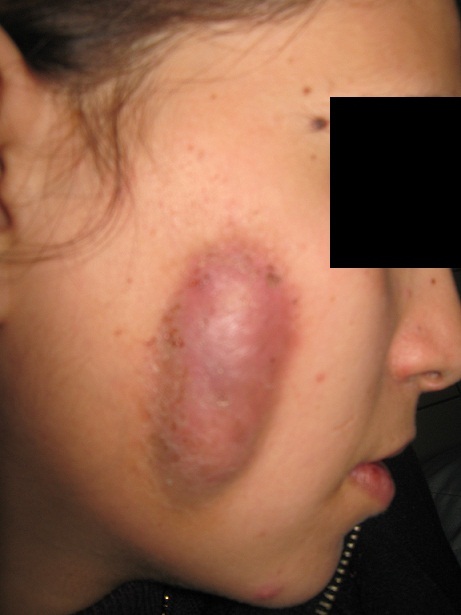
Tuméfaction sous-cutanée de la joue droite, grossièrement ovalaire, de couleur rouge cuivré, bien limitée et mesurant 5cm de grand axe

